# Sensors for Deformation Monitoring of Large Civil Infrastructures

**DOI:** 10.3390/s18113941

**Published:** 2018-11-14

**Authors:** Maria Marsella, Marco Scaioni

**Affiliations:** 1 Department of Civil, Environmental and Building Engineering (DICEA), Università degli Studi di Roma ‘La Sapienza’, 00184 Roma, Italy; maria.marsella@uniroma1.it; 2 Department of Architecture, Built Environment and Construction Engineering (DABC), Politecnico di Milano, 20133 Milano, Italy

**Keywords:** deformation, infrastructure, integrated monitoring system, measurement, monitoring, sensor network

In the maintenance of large infrastructures such as dams, bridges, railways, underground structures (tunnels, mines) and others, monitoring of deformations plays a key role in maintaining the safety serviceability conditions and for mitigating any consequences due to ageing factors and possible structural failures. This concern is, today, even more important than in the past due to the relevance of such infrastructures in our society, especially when they are related to communications and transportation.

This Special Issue on “Sensors for Deformation Monitoring of Large Civil Infrastructures” is aimed at providing the readers an up-to-date overview of the methodologies, technologies, and data processing techniques in this field. Fifteen articles are presented, including a review paper. Authors of those contributions come from ten countries, a large fraction of them from China.

Multiple techniques are described and presented in applications focused on different types of infrastructures. Very often, the corroborative and integrated use of more sensors units and sensor types is adopted. Indeed, the complexity that large infrastructures may feature calls for the use of multiple sensors organized in *integrated monitoring systems*. Signals recorded by different instruments can be conveyed to a control unit that can filter and evaluate all the outputs to feed numerical models able to predict the future behavior of the structure. On the sensor side, several new technologies have been developed and successfully applied in recent years. More and more often, the communication among sensors and with the control unit is based on wireless communications (*Wireless Sensor Networks*—WSNs).

A group of papers in this Special Issue deals with different types of *local geotechnical/structural deformation sensors* organized in *sensors networks*. In Zhao [1] the monitoring of potential ground surface deformations after back-filling of underground gob mines with waste rock is afforded. A network of sensors connected using the industrial Ethernet technology has been tested in a real case study in Hubei Province, China. This solution has been proved to be effective for detecting deformations just after the back-filling process and potentially in the long term. In addition, important information about the spatial and temporal distribution of deformations could be retrieved to have a better understanding of such a process, which may result in severe subsidence when the mine spans below urbanized areas, infrastructures or water bodies on surface. In Xu [2] an automatic and wireless *tunnel deformation* monitoring system using ultrasonic transducers is described. By processing the redundant ultrasonic information, the measurement accuracy can be improved under various probe angles, distances, and surrounding temperature variations. Ma [3] proposed a *Fiber Bragg Grating*-based dynamic tension detection system for overhead transmission line galloping, that may overcome some limitations of traditional monitoring methods adopted to this purpose. WSNs may also be used to detect other processes, as presented in Cheung [4]. This paper integrates *Building Information Modelling* (BIM [5]) techniques and WSNs into a unique system that enables us to visually monitor the safety status of an underground construction site, with specific attention posed on hazardous gases and environmental parameters (i.e., temperature and humidity). In addition, the application of BIM tools for the management of buildings is today largely popular, but their extension to the infrastructures still require much research and development work [6].

*Geotechnical/structural contact sensors* may yield continuous observations at key points of a structure, with the chance of also being embedded inside structural elements, such as in the case of fiber optic sensors. In this category, *robotic geodetic instruments* and *GNSS* sensors can provide 3D absolute displacements of a structure’s body [7]. On the other hand, *areal-based deformation measurement* [8] techniques have been developed to depict a general overview of the deformation pattern on the basis of *terrestrial remote sensors*, such as laser scanning and ground-based interferometric SAR systems.

A recurrent topic in several paper concerns the use of *terrestrial laser scanning* (TLS) instruments for deformation monitoring applications. Thanks to the acquisition of dense and redundant 3D point clouds or 2D profiles, this technique may be exploited to obtain information about changes over full surfaces rather than on a small number of control points as in traditional deformation measurement techniques [9]. Yang [10] developed a full cross-sectional laser scanner together with a visualization software package for the measurement of inner tunnel profiles. The developed system used a polar coordinate measuring method able to carry out the full cross-sectional measurement by using a rotating laser sensor. The potential impact of gateroad wall flatness, roughness, and geometrical profile, as well as the presence of coal dust have been studied. A TLS instrument was applied in Holst [11] for determining the corresponding changes of focal lengths and areal reflector deformations of a radio telescope. Indeed, these effects might occur during the rotation of the radio telescope’s structure. Their evaluation is thus needed to improve the accuracy of astronomical observations. Since deformations may be at millimeter level on a structure featuring a diameter of approximately 20 m, a procedure to minimize systematic measurement errors on the basis of a bundle adjustment including data from both sides of the radio telescope was proposed. Barbarella [12] proposed and tested a procedure for TLS data acquisition and a processing chart finalized to faulting identification and measurement on jointed concrete pavements in airports. The method is based on the normals’ computation after Least-Squares fitting of the entire pavement point cloud and at the level of each slab.

*Structural health monitoring* (SHM) of long-span bridges is today a hot-topic because of the plethora of such types of structures that have been constructed in recent years, and to the existence of several old ones that are still in full service. As demonstrated by the collapse of “Morandi” bridge in Genua (Italy) on 14 August 2018, a great attention should be paid to this problem either by technology developers and by officers in charge of SHM. In addition to the use of a wide range of in-situ monitoring instruments, Meng [13] present a feasibility study project (GeoSHM) that used the GNSS and satellite Earth Observation techniques in long-span bridges.

Dams and water barrages are other important types of infrastructures whose monitoring requires great attention. This special issue also hosts a review of geodetic and remote-sensing techniques for *dam deformation measurement* [14], which illustrates the state-of-the-art sensor technology in this field and draws future perspectives. In Barzaghi [15] the application of multiple GNSS sensors together with up-to-date processing techniques to measure the displacements of a dam structure is presented. Results obtained from 2.5-year observations have been compared to the ones obtained with a pendulum installed in the middle vertical section of the dam. After fitting time series with analytical models, sub-millimetric standard deviations of residuals have been found. This achievement demonstrates that GNSS technique may be profitably applied to dam monitoring allowing a denser spatial and temporal description of the dam displacements with respect to observations from pendulum. In Corsetti [16], the limited spatial coverage of traditional and GNSS deformation monitoring techniques for dam structures have been overcome by using microwave satellite-based technologies, such as *Advanced Differential Synthetic Aperture Radar Interferometry* (A-DInSAR). The major contribution of this paper is the exploitation of such a large number of observed point displacements, which are distributed along the whole structure and are characterized by millimetric accuracy on displacement rates, for the calibration of numerical models. These models are implemented to simulate the structural behavior of a dam under conditions of stress thus improving the ability to maintain safety standards.

A-DInSAR observations from satellites are becoming more and more reliable for wide area and long-term deformation monitoring. One of these techniques (namely *Persistent Scatterer Interferometry*—PSI [17]) has been also applied in Qin [18] for *subsidence* mapping over the entire transportation network of Shanghai (China) including elevated roads, ground highways and underground subways. The precise geolocation and structural characteristics of these infrastructures were combined to effectively guide a more accurate identification of the observed persistent scatterers, showing highly consistency in terms of subsidence velocities and time-series displacements.

Two papers [19,20] deal with the precise measurement of railway tracks, which is a task of fundamental importance to ensure the track quality in both the construction phase and the regular serviceability phases. Chen [19] proposed the integration of an *inertial navigation system* (INS) with geodetic instruments to set up a modular Railway Track Geometry Measuring Trolley system. This solution, which may be configured according to different surveying tasks including: precise adjustment of slab tracks, providing tamping measurements, measuring track deformation and irregularities, and determination of the track axis. Gabara and Savicki [20] proposed an image-based [21] measuring method for geometric parameters determination of a railway track. After processing using *Structure-from-Motion* (SfM) photogrammetry [22] and *dense surface matching* [23], the achieved results have been compared to those obtained from geodetic measurements. The achieved accuracy has met the normative requirements for measurement and inspection of the rail tracks.

The adoption of *photogrammetric/image-based* techniques [24] for measuring deformations was also pursued by Ong [25] for monitoring floating covers used in waste water treatment plants, which are one of the many structures formed with membranes. This paper proposed a potentially cost-effective non-contact approach for full-field strain and stress mapping using an *unmanned aerial vehicle* (UAV [26,27]) carrying a digital camera and a GNSS tracker. The images acquired by the UAV are then used to define the geometry of the floating cover using SfM Photogrammetry. This technique is also included in the areal-based deformation measurement techniques [8].

In the end of this Special Issue, both guest editors would like to thank all people who have contributed, including: All the authors of submitted papers, the reviewers who gave valuable support, and the Editorial Office of Sensors (MDPI). We hope that this Special Issue will further promote research on the sensor technology and applications in the field of deformation monitoring of large infrastructures. The authors also hope that the published contributions will foster the process of technology and knowledge transfer from the scientific community to public institutions and private companies who are in charge of maintaining the safety conditions of any types of infrastructures.
